# Polyvinyl alcohol-iodine induced corneal epithelial injury *in vivo* and its protection by topical rebamipide treatment

**DOI:** 10.1371/journal.pone.0208198

**Published:** 2018-11-29

**Authors:** Masamichi Fukuda, Shinsuke Shibata, Naoko Shibata, Nobuyuki Fujita, Hisanori Miyashita, Naoki Tanimura, Hidetoshi Ishida, Eri Kubo, Hiroshi Sasaki

**Affiliations:** Department of Ophthalmology, Kanazawa Medical University, Kahoku, Ishikawa, Japan; Cedars-Sinai Medical Center, UNITED STATES

## Abstract

Periocular povidone-iodine (PI) and polyvinyl alcohol-iodine (PAI) have had a major role in the prevention of endophthalmitis. The purpose of this study was to investigate the corneal epithelial toxicity of PAI in a rabbit eye model using corneal resistance (CR) measurement, which is a good indicator of cell barrier function. Rabbit eyes were administered PAI solution at 4-, 6-, 8-, or 16-fold dilution with physiological saline solution (saline) or saline alone (control), to the conjunctival sac with/without wash-out with saline. Corneal epithelial injury assessed by fluorescein staining and the CR ratio was measured at 10 minutes (min) to 96 hours (h) after the initial administration. Histological observation was performed in the eyes following the PAI or control administrations. At 120 min after administration of PAI solution, the CR ratio was decreased and superficial punctate keratopathy (SPK) was significantly increased in each of the PAI-administered groups compared to the control. Recovery of CR and SPK after administration of 6- or 8-fold dilution of PAI was significantly delayed in eyes that were not subsequently washed with saline compared with eyes that were. Pre- or post-instillation of 2% rebamipide ophthalmic suspension significantly reduced PAI induced-SPK and -decrease of CR ratio. The CR method was able to accurately and quantitatively evaluate fine corneal epithelial injury. It is suggested that eyes should be washed with saline solution after administration of PAI solution or the instillation of rebamipide to prevent or reduce corneal epithelial injury.

## Introduction

Endophthalmitis is a rare but potentially devastating complication of cataract surgery. In previous studies, rates of endophthalmitis after cataract surgery have ranged from 0.03% to 0.70% [1–3]. There is great variability in endophthalmitis prophylaxis practice patterns worldwide. Periocular povidone-iodine (PI) was once universally adopted and considered to be the standard of eye care in most practices. PI is most widely used as a preventive agent largely because of its strong bactericidal effects on many strains of bacteria, including drug-resistant bacteria, viruses, and other pathogens [4]. Ocular administration of eye drops containing 5% PI has been shown to reduce the number of bacteria and the risk of postoperative endophthalmitis [5, 6]. In addition, previous studies have shown that 5% PI is a safe sterilization agent on the ocular surface during ocular surgery [5–8]. On the other hand, several studies have reported that instillation of 2.5% and 5% PI into the conjunctival sac caused severe epithelial damage in the cornea [9]. In 2011, a notice from Meiji Seika Pharma, a Japanese pharmaceutical company whose PI preparation products are used in Japan, stated that PI can cause toxic and allergic reactions when used ophthalmically, and PI should not be used for ocular surface irrigation [10].

Fortunately, another generic iodine compound, polyvinyl alcohol-iodine (PAI), has been developed and approved for ophthalmic use in Japan. In this compound, iodine is combined with polyvinyl alcohol instead of polyvinylpyrrolidone as in PI [4]. In Japan, 85% of surgeons in 2014 reported using iodine compounds (36% PI and 49% PAI) for conjunctival irrigation before surgery [11]. It has been reported that PAI has a disinfectant capability similar to that of PI [10]. PAI is a broad-spectrum microbicide with high efficiency against bacteria, viruses, fungi and protozoans. The antimicrobial activity of PAI is derived from free iodine. In ophthalmic surgery, PAI is applied to the external eye immediately before intraocular surgery to reduce the incidence of endophthalmitis [12–14]. Moreover, treatment with PAI showed less toxicity than PI in a cultured human corneal epithelial cell line [15]. The current treatment recommendation consists of ocular surface irrigation of the operative field every 20 seconds with saline containing 0.025% PI or 0.0025% PAI during cataract surgery in order to achieve a very low bacterial contamination rate in the anterior chamber [11, 16, 17]. However, despite the widespread replacement of PI with PAI, it has not been determined whether frequent use of PAI during ocular surgery has toxic effects on the corneal epithelium. Indeed, there have been few reports on the safety or toxicity of PAI in corneal epithelial cells *in vivo*. Frequent use of PAI during surgery may affect the patients with potential damage to the ocular surface such as dry eye, diabetic and contact lens wearer so on. Thus, the evaluation of toxicity or safety of PAI treatment during eye surgery should be examined *in vivo*. Furthermore, the prevention of PAI-induced toxicity in cornea should be performed during eye surgery. Rebamipide (Otsuka Pharmaceutical Co., Ltd., Tokyo, Japan) is a mucosal protective agent and is marketed in Japan as an oral drug for the treatment of gastric mucosal disorders since 1990. Rebamipide is a quinolinone derivative that induces production and secretion of mucin-like substances in the cornea and conjunctiva [18–21]. Rebamipide promotes the healing of corneal injuries and increases tear film stability in patients with dry eye [18, 20, 22]. Currently, rebamipide ophthalmic suspension has been used for the treatment of dry eye since 2012 in Japan. We have shown that rebamipide ophthalmic suspension was effective in treating keratoconjunctivitis sicca of patients with Sjögren syndrome, probably by increasing mucins and suppressing inflammatory cytokines [18]. In the present study, therefore, we investigated PAI-induced corneal epithelial injury by measuring the corneal resistance (CR) and superficial punctate keratopathy (SPK) following PAI administration in an *in vivo* rabbit eye model.

In this study, we investigated the protection of PAI-induced corneal damage by the wash-out of PAI using physiological saline or the topical administration of 2% rebamipide ophthalmic suspension.

## Materials and methods

### Reagent

PAI solution “PA-IODO Ophthalmic and Eye washing Solution” (Nitten Pharmaceutical Co., Ltd., Nagoya, Japan), which has an effective iodine concentration of 0.2% before dilution, was diluted 4-fold (0.05%), 6-fold (0.033%), 8-fold (0.025%), or 16-fold (0.0125%) with saline (Otsuka Normal Saline isotonic 0.9% sodium chloride solution; Otsuka Pharmaceutical). Twenty mL of these dilutions or saline alone was infused into rabbit eyes with or without 20-mL saline wash-out. 2% rebamipide (2-(4-chlorobenzoylamino)-3-[2(1H)-quinolinon-4yl-]-propionic acid) ophthalmic suspension (rebamipide) was provided by Otsuka Pharmaceutical.

### Animals

This study was carried out in strict accordance with the recommendations in the Guide for the Care and Use of Laboratory Animals of the National Institutes of Health. The protocol was approved by the Institutional Review Board on the Use of Animals at Kanazawa Medical University (Protocol Number: 2017–63). All surgery was performed under the instillation of Oxybuprocaine Hydrochloride eye drop (Benoxil Ophthalmic solution, Santen Pharmaceutical Co., Ltd., Tokyo, Japan) and all measures were taken to minimize suffering. The animals comprised 36 adult male albino New Zealand white rabbits, and both eyes of each rabbit were studied (age: 6.53±0.93 weeks old; body weight: 3.0–3.5 kg). The animals were purchased from Sankyo Labo Service Corporation (Toyama, Japan) and kept under natural light-dark cycle in cages with ad libitum access to food and water in a room maintained at a temperature of 23°C. Animals of all subgroups were killed 1 day after the experiment by intravenous injection of sodium pentobarbital somnopentyl 64.8 mg/kg body weight (Kyoritsu Seiyaku Corp, Tokyo, Japan).

### Fluorescein corneal staining and classification of SPK Grade in the rabbit

Corneal staining with sodium fluorescein was performed to grade SPK. After fluorescein corneal staining, the total sum of the area of SPK was graded from A0 through A3, and the density was graded from D0 through D3 as an Area and Density (AD) classification following the previous report.[19] using an SL-130 slit-lamp microscope (Carl Zeiss Meditec, Tokyo, Japan).

### Measurement of CR

To estimate corneal barrier function, we have established the measurement of corneal resistance (CR) [20–22]. This CR device enables the quantitative assessment of corneal disease *in vivo*, to measure the degree of corneal injury with reliable reproducible data. Corneal injury was quantified by CR measured by a newly improved electrical CR device (Fukuda Model 2015), which was combined with a vaginal impedance reader (Model MK-10C; Muromachi Kikai, Osaka, Japan) and a corneal contact lens electrode (Mayo Corporation, Aichi, Japan) [12]. The ratio of post-treatment CR to baseline CR was defined as the CR ratio (%).

### Histological examination

Corneal tissues were isolated from the harvested eye tissues. The collected corneal tissues were fixed for 24 h in 4% paraformaldehyde, embedded in paraffin and sectioned at approximately 4 μm. The paraffin sections were stained with hematoxylin-eosin (H&E) and processed for histopathological examination.

### PAI infusion

Thirty eyes of 15 rabbits were divided into 5 groups (6 eyes per group): 4 groups treated with PAI solution (4-, 6-, 8-, or 16-fold dilution with saline) and 1 group administered saline solution alone (control).

First, all eyes were examined with fluorescein staining to ensure absence of injury and baseline measurements of CR were made. Ten min later, 20 mL of PAI or saline was administered to the conjunctival sac using a syringe without a needle, and 10 seconds after these treatments the eyes were washed with 20 mL of saline applied in the same way. Corneal epithelial damage was assessed by FL and CR at 10, 30, 60, and 120 minutes (min) after the initial administration. Animals of all subgroups were killed 1 day after the procedure and eyes were enucleated and processed for histological observation.

### Saline washing procedure

Twenty-four eyes of 12 rabbits were divided into 4 groups (6 eyes per group). PAI diluted 6- or 8-fold with saline was administered using a similar method as described in the above section, then, 10 seconds later, the eyes were irrigated with saline or left unwashed. The CR ratio was measured with a CR device at 10, 30, 60 and 120 min and 24, 48, 72, and 96 h after the initial administration.

### Treatment f of rebamipide ophthalmic suspension

Eighteen eyes of 9 rabbits were divided into 3 groups (6 eyes per group). PAI diluted 8-fold with saline was administered using a similar method as described in the above section, then, 10 seconds later, the eyes were irrigated with saline. In pre-operative instillation group, 30 minutes before treatment with PAI diluted 8-fold with saline, rebamipide was instilled every 5 minutes for 5 times. In postoperative instillation group, rebamipide was instilled 1 hour after irrigating eyes with saline. The instillations of rebamipide were performed 3 times a day for 72 hours. In control group, physiological saline solution was instilled as with pre- and post-instillation group. CR ratio was measured with a CR device at 0, 1, 24, 48, 72, and 96 h after the initial administration.

### Statistical analysis

For all quantitative data collected, statistical analysis was conducted by Student’s t test and/or one-way ANOVA when appropriate, and was presented as mean ± S.D. of the indicated number of experiments. A significant difference between control and treatment group was defined as p-value of < 0.05 for six eyes.

## Results

### Effect of PAI Solution on the corneal epithelium

**Fig 1:**
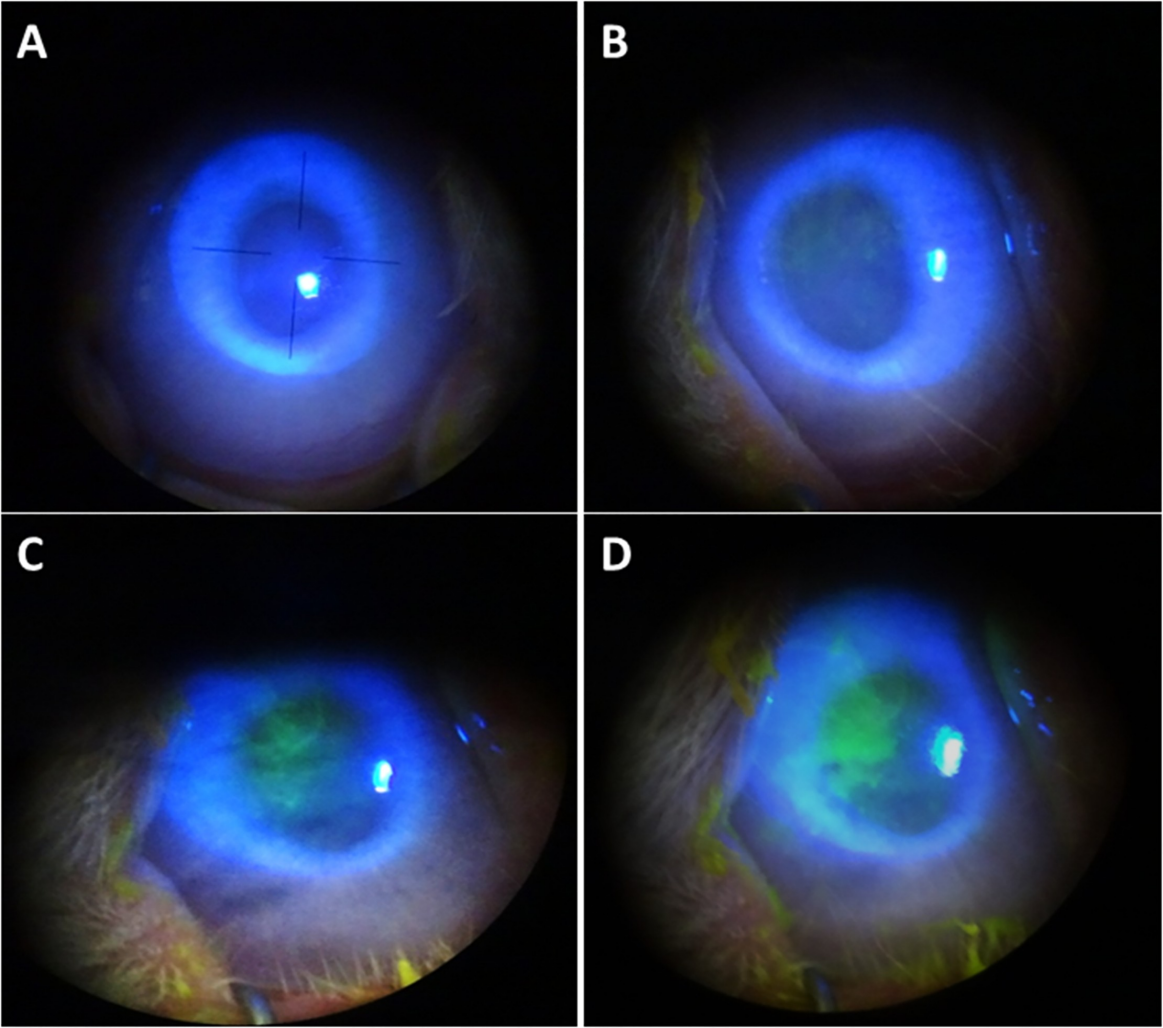
Clinical evaluation of effect of PAI on the ocular surface. PAI solution diluted 6-, 8-, or 16-fold with saline and saline solution as a control were administered to rabbit eyes, followed by washing with saline 10 seconds later. Corneal epithelial damage was assessed by fluorescein staining. (A) Representative images of fluorescein corneal staining showed no substantial staining in the saline controls. (B) Representative images of fluorescein staining showed mild diffuse corneal staining at the central part of cornea in the 16-fold PAI-treated eyes with no obvious staining in the untreated controls, (C) mild diffuse fluorescein staining at the central part of cornea in all 8-fold PAI-treated eyes, and (D) patches and diffuse staining at the central part of cornea in 6-fold PAI-treated eyes.

Corneal epithelial damage after PAI treatment was detected by FL and graded by observation with a cobalt blue filter under a slit-lamp microscope (Fig 1). At 120 min after administration, only the saline control group was negative for fluorescein staining, whereas every PAI dilution group was positive, with the 4-fold dilution showing the greatest effect and highest AD grade, followed by the 6-, 8- and 16-fold groups (Fig 1).

By AD classification of fluorescein staining, the grade of corneal epithelial injury 120 min after administration was A2D2 (6/6 eyes) in the 4-fold group, A1D1 (3/6 eyes) and A2D2 (3/6 eyes) in the 6-fold group, A1D1 (6/6 eyes) in the 8-fold group, A0D0 (1/6 eyes) and A1D1 (5/6 eyes) in the 16-fold group, and A0D0 (6/6 eyes) in the saline control group (Table 1).

**Table 1:** AD classification of fluorescein corneal staining (FL) and CR ratio (%) in rabbit eyes administered 4-, 6-, 8-, 16-fold diluted PAI solution, or saline as a control.

120 minutes after administration		AD classification of FL: Eyes	CR (%)
PAI solution	4-fold	• A2D2 : 6	65.8
	6-fold	• A2D2 : 3 • A1D1 : 3	71.2
	8-fold	• A1D1 : 6	77.4
	16-fold	• A1D1 : 5 • A0D0 : 1	83.6
Saline (control)		• A0D0 : 6	99.2

By AD classification, fluorescein staining was negative in all eyes in the control group, whereas all PAI-treated eyes were positive for fluorescein staining at 120 min after administration, with the level of positivity being concentration-dependent. The CR ratios were also increased in a dose-dependent manner.

FL: fluorescein corneal staining, AD: area and density, CR: corneal resistance ratio (post-treatment/pre-treatment), PAI: polyvinyl alcohol-iodine

**Fig 2:**
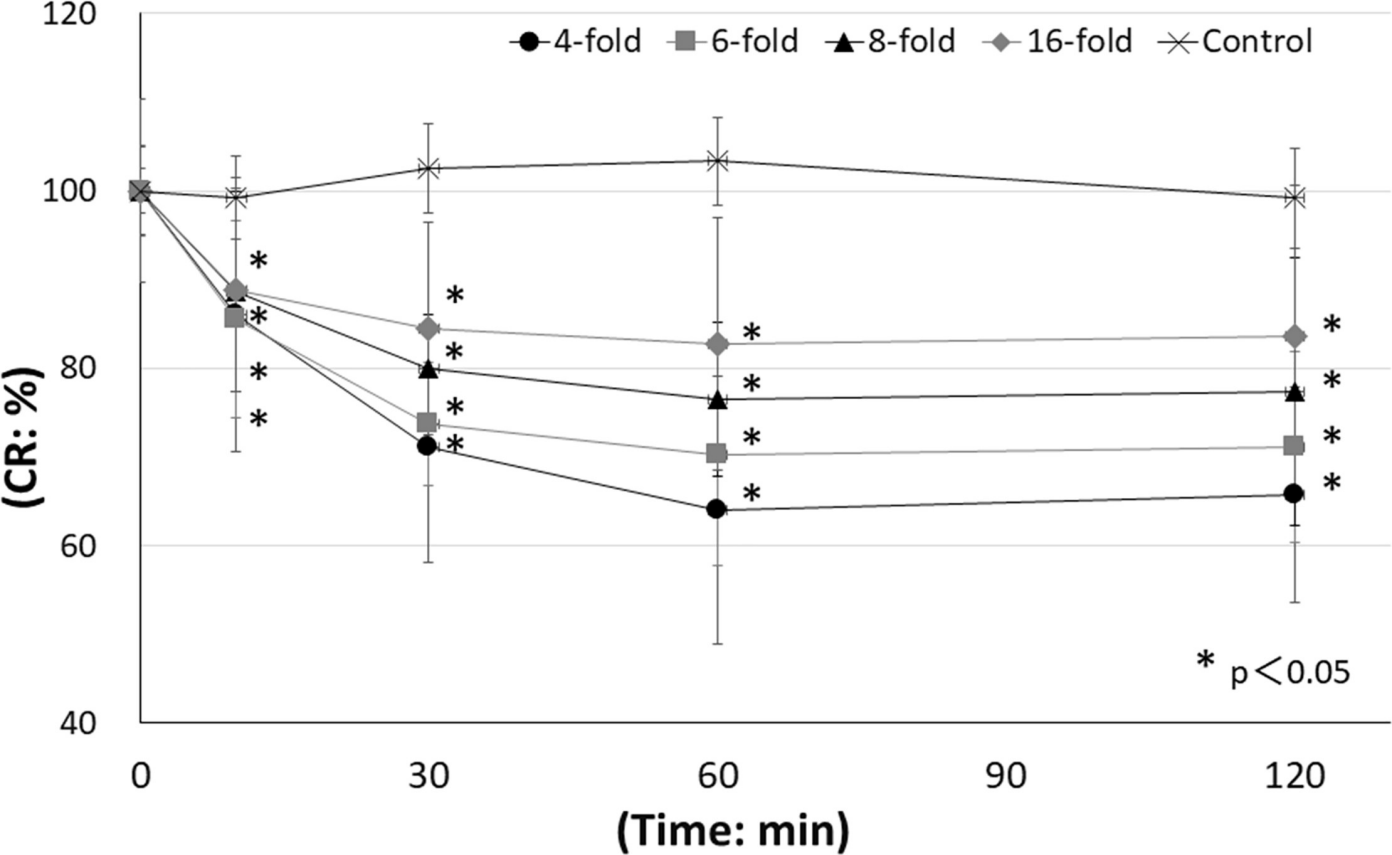
Time course of CR ratio following administration of saline or PAI. PAI solution diluted 4-, 6-, 8-, or 16-fold with saline and saline solution as a control were administered to rabbit eyes, followed by washing with saline 10 seconds later. At 0, 10, 30, 60, and 120 min after the initial administration, corneal epithelial damage was measured by a CR device. The CR ratio was decreased in a time- and dose-dependent manner. ●: 4-fold group, ■: 6-fold group, ▲: 8-fold group, ◆: 16-fold group, X: Saline control group. *p<0.05 versus control, N = 6.

The CR ratios after 120 min were 65.8±12.2%, 71.2±10.7%, 77.4±15.1%, 83.6±16.9% and 99.2±5.6% in the 4-, 6-, 8- and 16-fold dilution groups and the saline control group, respectively. CR ratios at 120 min in each concentration group were significantly different from the CR ratio in the controls (p<0.05 versus control) (Fig 2).

**Fig 3:**
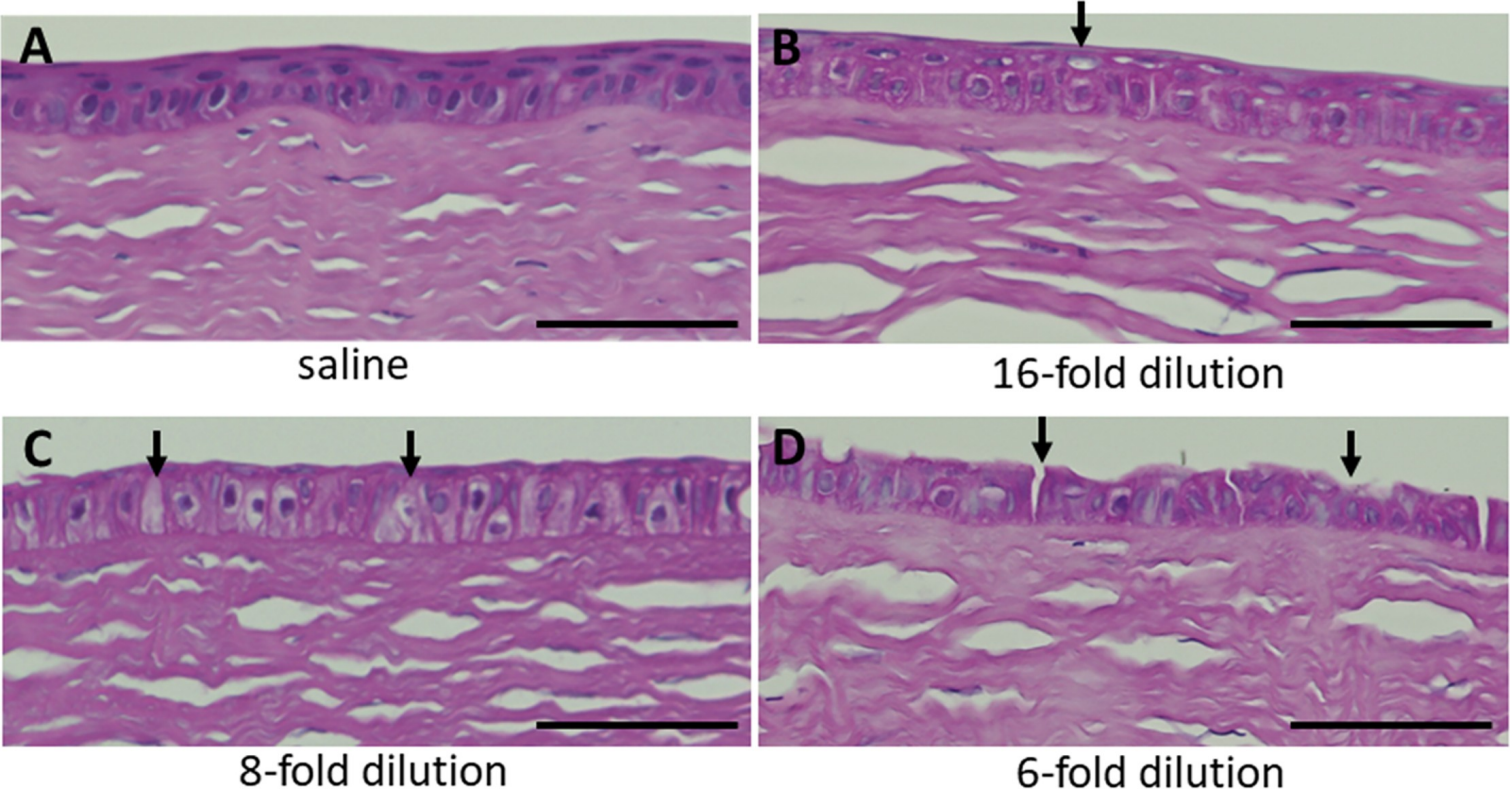
Histological observation of the rabbit cornea after treatment with 6-, 8- and 16-fold diluted PAI. PAI solution diluted 6-, 8-, or 16-fold with saline and saline solution as the control were administered to rabbit eyes, followed by washing with saline 10 seconds later. Corneal epithelial damage was observed by staining with H&E. (A) In the controls treated with normal saline, no corneal damage was observed. (B) In the 16-fold treatment group, a superficial epithelial layer was observed. Arrows indicate damage to surface cells. (C) In the 8-fold treatment group, thinning of the superficial layer was apparent. Arrows indicate impairment of the surface epithelium. (D) In the 6-fold treatment group, loss of the superficial epithelium and some epithelial cells was observed. Arrows indicate impairment of surface cells. Bar = 50μm.

To quantify corneal damage after PAI treatment, we performed histological analysis by H&E staining at 120 min after administration (Fig 3). Sectioned corneal tissues treated with normal saline and 6-, 8- and 16-fold PAI were examined for morphological changes, and no apparent abnormalities were detected in the rabbit corneas treated with normal saline (Fig 3A). In the 16-fold treatment group, slight epithelial damage was found in the superficial layer of the central part of corneal epithelium (Fig 3B). In the 8-fold treatment group, the superficial cell layer appeared to be thinner at the central part of corneal epithelium (Fig 3C). In the 6-fold treatment group, desquamation of the outermost layer, an irregular surface of the basal cells and further loss of epithelial cells were observed at the central part of corneal epithelium (Fig 3D). In all groups, PAI did not affect other corneal layers such as stroma and endothelium. Limbal parts of cornea was not affected by PAI treatment.

### Effect of wash-out with physiological saline on PAI-induced corneal epithelial injury

By AD classification, positive findings were observed in all eyes exposed to 6- or 8-fold dilution in the non-washed group (Table 2). In contrast, in the washed group, fluorescein staining was not observed in most eyes exposed to 6- or 8-fold dilution.

**Table 2:** AD classification of FL and CR ratio (%) in eyes administered 6- or 8-fold diluted PAI solution with/without wash-out by saline solution.

	PAI 6-fold dilution				PAI 8-fold dilution			
	Non-washed		Washed		Non-washed		Washed	
Hours after administration	AD classification of FL: eyes	CR (%)	AD classification of FL: eyes	CR (%)	AD classification of FL: eyes	CR (%)	AD classification of FL: eyes	CR (%)
0	• A0D0 : 6	100	• A0D0 : 6	100	• A0D0 : 6	100	• A0D0 : 6	100
24	• A1D1 : 4 • A1D2 : 1 • A2D1 : 1	67.2	• A1D1 : 5 • A2D2 : 1	88.1	• A0D0 : 1 • A1D1 : 3 • A2D2 : 2	65.9	• A0D0 : 5 A1D1 : 1	78.1
48	• A1D1 : 6	67.2	• A0D0 : 1 • A0D1 : 1 • A1D0 : 1 • A1D1 : 3	91.1	• A0D0 : 2 • A1D1 : 4	69.1	• A0D0 : 6	89.5
72	• A1D0 : 1 • A1D1 : 4 • A2D1 : 1	73.3	• A0D0 : 3 • A1D1 : 2 • A2D2 : 1	96.6	• A0D0 : 2 • A1D1 : 4	78.0	• A0D0 : 6	100
96	• A1D1 : 6	77.5	• A0D0 : 4 • A1D1 : 2	103.4	• A0D0 : 5 • A1D1 : 1	90.2	• A0D0 : 6	100

PAI: polyvinyl alcohol-iodine, FL: fluorescein staining, AD: area and density, CR: corneal resistance ratio (post-treatment/pre-treatment)

**Fig 4:**
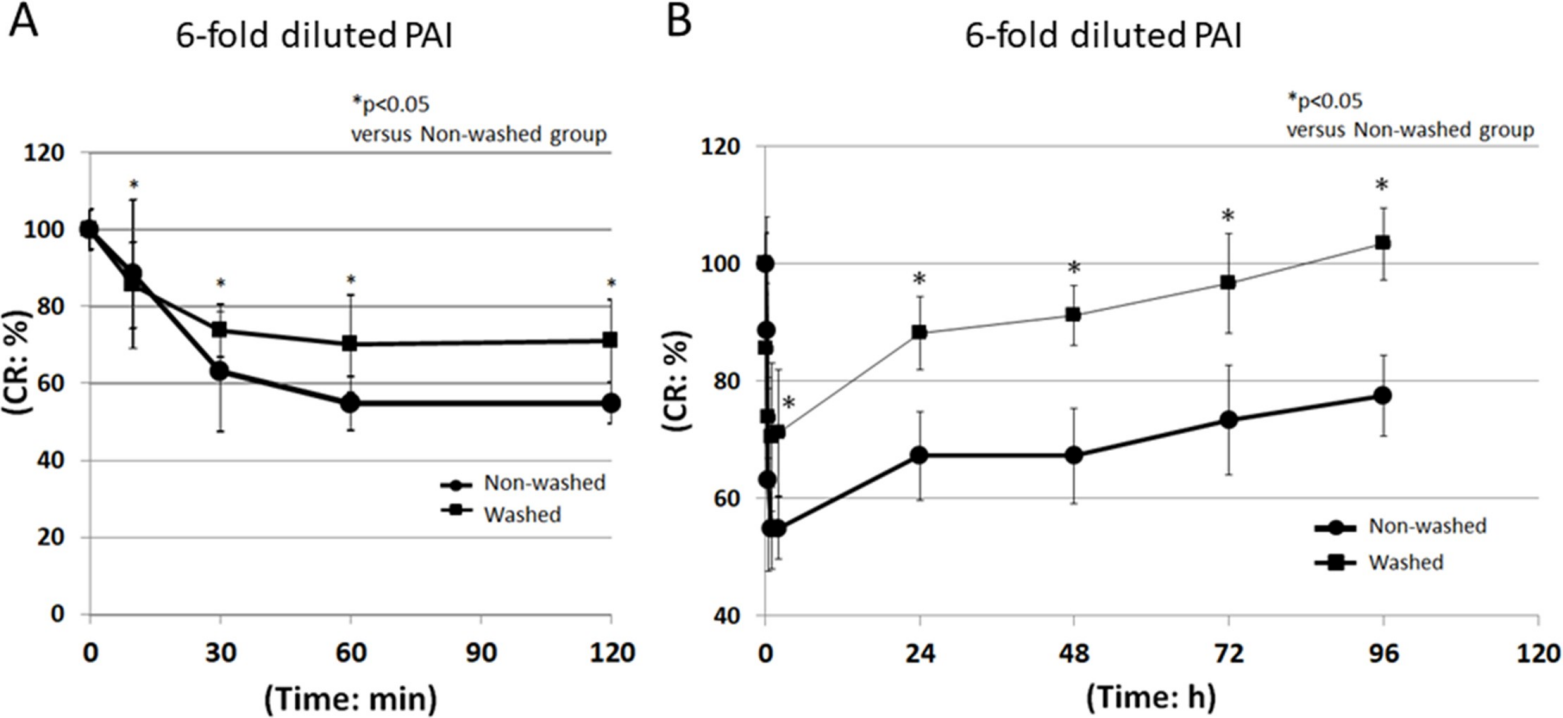
CR ratio following administration of 6-fold diluted PAI. PAI solution diluted 6-fold with saline was administered to rabbit eyes. Ten seconds later, the eyes were irrigated with saline or left unwashed. At 0, 10, 30, 60, and 120 min (A), and 0, 30, 60, and 96 h (B) after the initial administration, the CR ratio was measured using a CR device. ●: Non-washed, ■: Washed, *p<0.05 versus control, N = 6.

**Fig 5:**
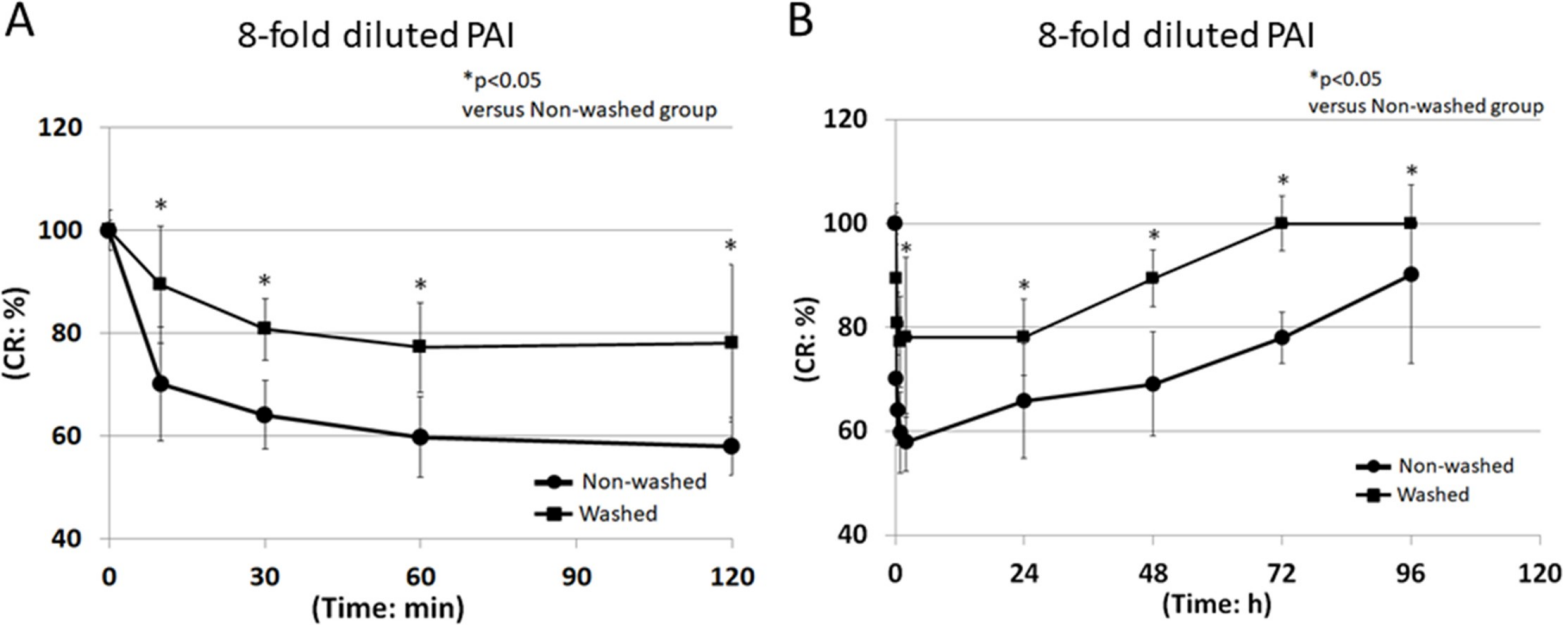
CR ratio following administration of 8-fold diluted PAI. PAI solution diluted 8-fold with saline was administered to rabbit eyes. Ten seconds later, the eyes were irrigated with saline or left unwashed. At 0, 10, 30, 60, and 120 min (A), and 0, 30, 60, and 96 h (B) after the initial administration, the CR ratio was measured using a CR device. ●: Non-washed, ■: Washed, *p<0.05 versus control, N = 6.

In the 6-fold group, the CR ratios at 120 min and 96 h in the washed group were 71.2±10.7% and 103.4±6.2%, and those in the non-washed group were 54.9±5.3% and 77.5±6.9%, respectively (Fig 4). In the 8-fold group, the CR ratios at 120 min and 96 h in the washed group were 78.1±15.4% and 100.0±0.0%, and those in the non-washed group were 57.9±5.6% and 90.2±17.2%, respectively (Fig 5). Recovery from 6- and 8-fold diluted PAI-induced corneal injury in the non-washed group was significantly delayed compared to the washed group at each time after initial administration of PAI (p<0.05) (Figs 4 and 5).

### Effect of rebamipide for PAI-induced corneal epithelial injury

**Fig 6:**
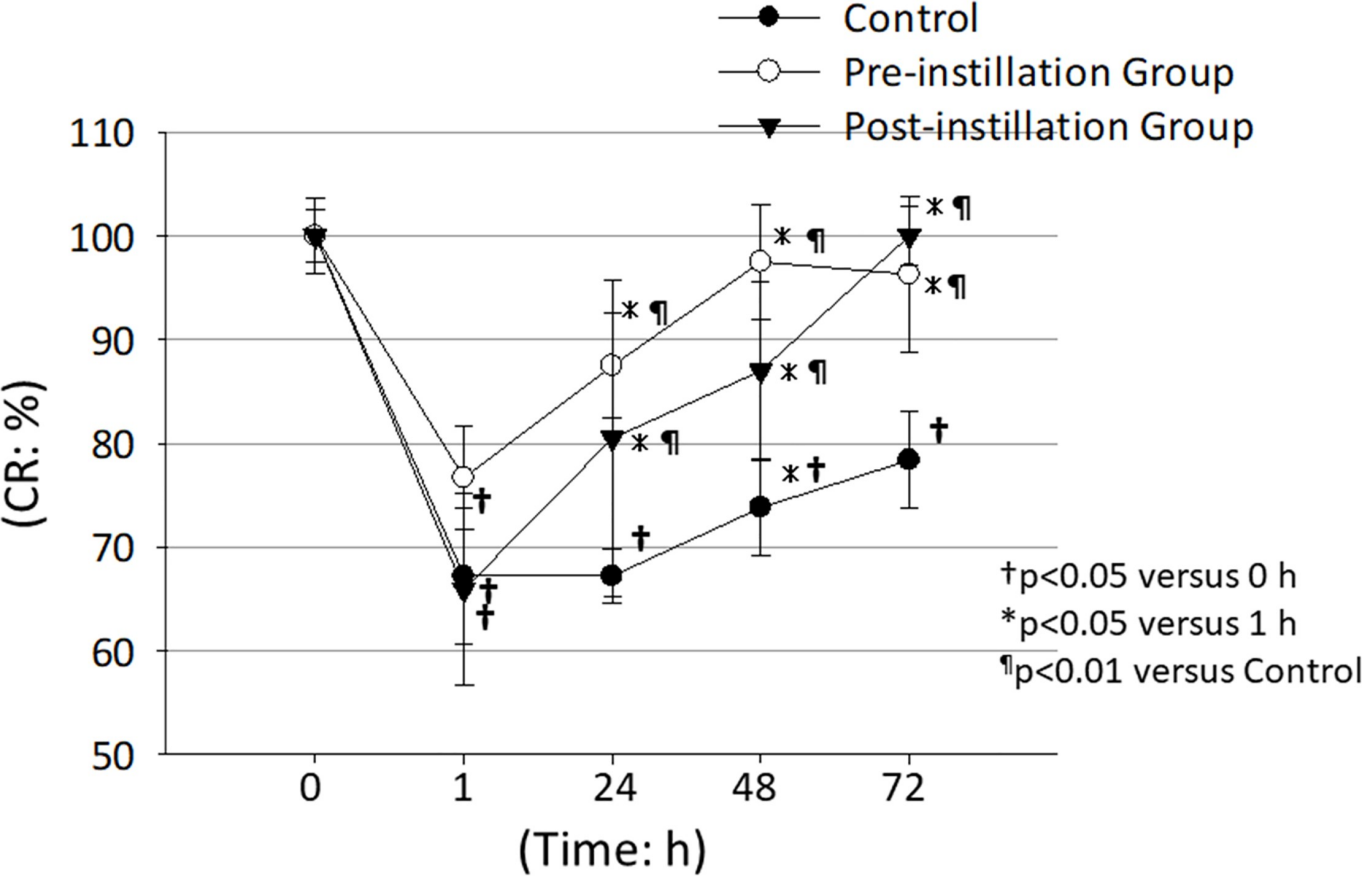
Effect of 2% rebamipide ophthalmic suspension on PAI-induced corneal damage. The CR ratios in the pre- and post rebamipide instillation groups were significantly higher than that in the control group suggesting rebamipide promoted the recovery of PAI-induced corneal damage. ●: Control group, ○: Pre-operative instillation group, ▼: Post-operative instillation group. †p<0.05 versus 0 h at each group, *p<0.05 versus 1 h at each group, ¶p<0.01 versus control group, N = 6.

In all groups, at 1h after the initial administration of 8-fold diluted PAI, CR ratio was significantly decreased compared to it at 0 h (†p<0.05) (Fig 6). In control group, the CR ratios at 1, 24, 48 and 72 h after the initial administration of 8-fold diluted PAI, CR ratio was significantly decreased compared to them at 0 h (†p<0.05) (Fig 6). In control group, the CR ratios at 72 h after the initial administration was significantly recovered compared to them at 1 h (*p<0.05) (Fig 6). On the other hand, in preoperative instillation group, the CR ratio at 48 and 72 h after the initial administration was significantly increased compared to them at 1 h (*p<0.05) (Fig 6). In postoperative instillation group, the CR ratio at 72 h after the initial administration was significantly increased compared to them at 1 h (*p<0.05) (Fig 6). In pre-instillation groups, the CR ratios at 24, 48 and 72 h were significantly increased compared to the control group (^¶^p<0.01) (Fig 6). In post-instillation group, CR ratios at 48 and 72 h were significantly increased compared to the control group (^¶^p<0.01) (Fig 6). These results suggest that the pre- or post-instillation of rebamipide is effective to protect the PAI-induced corneal damage.

## Discussion

In the present study, we reported first time that PAI induces corneal epithelial injury in an *in vivo* rabbit eye model. In particular, the damage to corneal epithelial cells exposed to 6- and 8-fold dilution of PAI was examined. In Japan, 6- and 8-fold dilutions of PAI are commonly used to minimize bacterial contamination of the anterior chamber during intraocular surgery [10]. Our results obtained by CR device clearly showed that corneal epithelial damage occurred in rabbit eyes exposed to PAI, even at a 16-fold dilution. In addition, it was confirmed that epithelial damage was severe in the non-washed group after the treatment with PAI. The recovery from PAI-induced corneal damage in the non-washed group was slow in comparison to that in the washed group. In other words, our results suggested that longer exposure to PAI solution resulted in greater corneal epithelial damage. The results from these experiments on rabbit eyes cannot be directly applied to humans; however, we believe they are informative when considering procedures used in human eyes.

Chou *et al*. [4] showed that PI has immediate cytotoxic and fixation effects on cultured corneal epithelial and stromal cells, suggesting that prolonged contact of PI with viable uncontaminated tissue should be avoided. Prolonged exposure to PI of the corneal surface in the presence of a corneal epithelial defect might result in keratocyte death with subsequent persistent epithelial defects, disrupting the keratocyte‒epithelial interaction, which might subsequently retard corneal re-epithelialization. Moreover, according to another report, PI can induce cytotoxicity at concentrations ranging from 5% to 10% *in vivo* and 0.08% to 0.32% *in vitro* [20]. Washing rabbit eyes with 1.25% PI results in superficial punctate keratitis [23]. These previous data strongly support our results. Although the mechanism of corneal epithelial damage by PAI is unclear, it has been reported that iodine in a different solution, PI, induced the inhibition of human corneal fibroblast and epithelial cell growth and repair via the oxidation and disruption of cell membrane integrity [4]. In addition, since PAI oxidatively modifies the membrane protein on the surface of the microorganism and sterilizes it in a short time, corneal epithelial injuries by PAI are also presumed to be caused by oxidation reaction.

In this study, we found that PAI induced decrease of CR ratio was significantly improved with the pre -and post-instillation of the rebamipide ophthalmic suspension compared with the control group. Rebamipide suspension is known to increase gastric endogenous prostaglandin E2 and I2, to promote the secretion of goblet cell mucins [24, 25] and to have other anti-inflammatory action [26]. The therapeutic effects of rebamipide may be due to its ability to increase corneal and conjunctival mucin-like substances and improve PAI-induced corneal injury *in vivo*. In addition, anti-inflammatory effects may also increase the barrier function and improve CR ratio against PAI-induced corneal damage *in vivo*.

To evaluate and quantify the barrier function of the corneal epithelium, an anterior slit-lamp fluorophotometer has usually been used in a clinical setting [27]. However, this device takes more than 30 min to take a single measurement. We previously developed an electrophysiologic method that uses corneal impedance measurement to quantify corneal barrier function or damage [20, 23, 28]. This method, the CR measurement, has been shown to accurately and quantitatively evaluate fine corneal barrier function not detected by the fluorescein staining method in previous studies using rabbit eyes [12, 22, 28]. We also previously found that the CR values in human healthy corneas were not altered by age, gender or corneal thickness; however, the CR values were decreased in injured human cornea epithelial cells staining fluorescein-positive [21]. Thus, CR levels may directly reflect corneal epithelial damage. CR measurement may thus be a safe and useful method to evaluate and quantify the corneal barrier function and corneal damage in rabbit and human eyes.

In conclusion, the present study demonstrated that irrigation with 4- to 16-fold PAI solution induces corneal epithelial damage in rabbit eyes, as evidenced by fluorescein staining and the CR ratio. Importantly, washing with saline solution not only alleviates PAI-induced corneal epithelial damage but also accelerates recovery from damage. Use of PAI solution at the 6- and 8-fold dilutions may be clinically useful to reduce the number of bacteria and the risk of postoperative endophthalmitis; however, additional procedures such as washing with saline solution or other topical treatment should be considered to reduce PAI-induced corneal epithelial damage.

## References

[1] Haripriya A, Baam ZR, Chang DF. Endophthalmitis Prophylaxis for Cataract Surgery. Asia Pac J Ophthalmol (Phila). 2017;6(4):324–9. doi: 10.22608/APO.2017200 . 28780782 10.22608/APO.2017200

[2] Sandvig KU, Dannevig L. Postoperative endophthalmitis: establishment and results of a national registry. J Cataract Refract Surg. 2003;29(7):1273–80. . 12900232 10.1016/s0886-3350(02)02048-5

[3] Taban M, Behrens A, Newcomb RL, Nobe MY, Saedi G, Sweet PM, et al Acute endophthalmitis following cataract surgery: a systematic review of the literature. Arch Ophthalmol. 2005;123(5):613–20. 10.1001/archopht.123.5.613 . 15883279 10.1001/archopht.123.5.613

[4] Chou SF, Lin CH, Chang SW. Povidone-iodine application induces corneal cell death through fixation. Br J Ophthalmol. 2011;95(2):277–83. 10.1136/bjo.2010.189407 . 21036788 10.1136/bjo.2010.189407

[5] Apt L, Isenberg S, Yoshimori R, Paez JH. Chemical preparation of the eye in ophthalmic surgery. III. Effect of povidone-iodine on the conjunctiva. Arch Ophthalmol. 1984;102(5):728–9. Epub 1984/05/01. . 6721765 10.1001/archopht.1984.01040030584025

[6] Speaker MG, Menikoff JA. Prophylaxis of endophthalmitis with topical povidone-iodine. Ophthalmology. 1991;98(12):1769–75. Epub 1991/12/01. . 1775308 10.1016/s0161-6420(91)32052-9

[7] Alp BN, Elibol O, Sargon MF, Aslan OS, Yanyali A, Karabas L, et al The effect of povidone iodine on the corneal endothelium. Cornea. 2000;19(4):546–50. Epub 2000/08/06. . 10928775 10.1097/00003226-200007000-00028

[8] Apt L, Isenberg SJ, Yoshimori R, Spierer A. Outpatient topical use of povidone-iodine in preparing the eye for surgery. Ophthalmology. 1989;96(3):289–92. Epub 1989/03/01. . 2652027 10.1016/s0161-6420(89)32897-1

[9] Jiang J, Wu M, Shen T. The toxic effect of different concentrations of povidone iodine on the rabbit's cornea. Cutan Ocul Toxicol. 2009;28(3):119–24. Epub 2009/08/22. 10.1080/15569520903080511 . 19694608 10.1080/15569520903080511

[10] Inoue Y, Usui M, Ohashi Y, Shiota H, Yamazaki T. Preoperative disinfection of the conjunctival sac with antibiotics and iodine compounds: a prospective randomized multicenter study. Jpn J Ophthalmol. 2008;52(3):151–61. Epub 2008/07/29. 10.1007/s10384-008-0517-y . 18661264 10.1007/s10384-008-0517-y

[11] Matsuura K, Mori T, Miyamoto T, Suto C, Saeki Y, Tanaka S, et al Survey of Japanese ophthalmic surgeons regarding perioperative disinfection and antibiotic prophylaxis in cataract surgery. Clin Ophthalmol. 2014;8:2013–8. Epub 2014/10/11. 10.2147/OPTH.S64756 ; PubMed Central PMCID: PMCPMC4189719. 25302013 10.2147/OPTH.S64756 PMC4189719

[12] Fukuda M, Takeda N, Shibata S, Shibata N, Shibata T, Sugiyama K, et al In vitro and in vivo corneal effects of latanoprost combined with brimonidine, timolol, dorzolamide, or brinzolamide. Eur J Pharmacol. 2016;787:43–6. Epub 2016/05/18. 10.1016/j.ejphar.2016.05.012 . 27181069 10.1016/j.ejphar.2016.05.012

[13] Hatano H, Sakamoto M, Hayashi K, Kamiya S. [Antimicrobial Effects of Iodine-Polyvinyl Alcohol Ophthalmic and Eye Washing Solution (PA * IODO) with Special Reference to its Temperature, Concentration and Time and its Preservation Stability]. Nippon Ganka Gakkai Zasshi. 2015;119(8):503–10. . 26390575

[14] Matsuura K, Terasaka Y, Sasaki S, Inoue Y. Assessment of Corneal Epithelial and Endothelial Cell Damage after Intraoperative Disinfection Using Occasional Polyvinyl Alcohol Iodine. Japanese Journal of Ophthalmic Surgery. 2014;27(3):451–5.

[15] Shibata Y, Tanaka Y, Tomita T, Taogoshi T, Kimura Y, Chikama T, et al Evaluation of corneal damage caused by iodine preparations using human corneal epithelial cells. Jpn J Ophthalmol. 2014;58(6):522–7. Epub 2014/09/19. 10.1007/s10384-014-0348-y . 25230910 10.1007/s10384-014-0348-y

[16] Shimada H, Arai S, Nakashizuka H, Hattori T, Yuzawa M. Reduced anterior chamber contamination by frequent surface irrigation with diluted iodine solutions during cataract surgery. Acta Ophthalmol. 2017;95(5):e373–e8. Epub 2017/03/09. 10.1111/aos.13390 . 28271619 10.1111/aos.13390

[17] Shimada H, Nakashizuka H, Grzybowski A. Prevention and Treatment of Postoperative Endophthalmitis Using Povidone-Iodine. Curr Pharm Des. 2017;23(4):574–85. Epub 2016/12/06. 10.2174/1381612822666161205105404 . 27917719 10.2174/1381612822666161205105404

[18] Arimoto A, Kitagawa K, Mita N, Takahashi Y, Shibuya E, Sasaki H. Effect of rebamipide ophthalmic suspension on signs and symptoms of keratoconjunctivitis sicca in Sjogren syndrome patients with or without punctal occlusions. Cornea. 2014;33(8):806–11. Epub 2014/07/01. 10.1097/ICO.0000000000000155 . 24977983 10.1097/ICO.0000000000000155

[19] Miyata K, Amano S, Sawa M, Nishida T. A novel grading method for superficial punctate keratopathy magnitude and its correlation with corneal epithelial permeability. Arch Ophthalmol. 2003;121(11):1537–9. Epub 2003/11/12. 10.1001/archopht.121.11.1537 [pii]. . 14609908 10.1001/archopht.121.11.1537

[20] Fukuda M, Sasaki H. Quantitative evaluation of corneal epithelial injury caused by n-heptanol using a corneal resistance measuring device in vivo. Clin Ophthalmol. 2012;6:585–93. Epub 2012/05/04. 10.2147/OPTH.S30935 [pii]. ; PubMed Central PMCID: PMC3340121. 22553418 10.2147/OPTH.S30935 PMC3340121

[21] Fukuda M, Sasaki H. In Vivo Measurement of Human Corneal Impedance Value. Cornea. 2016;35(10):1305–7. Epub 2016/05/27. 10.1097/ICO.0000000000000894 . 27227395 10.1097/ICO.0000000000000894

[22] Fukuda M, Yamamoto K, Takahashi N, Sasaki H, Yoshikawa M. Corneal disorder quantification by corneal resistance. Atarashii Ganka (Journal of the Eye). 2007;24(4):521–5. .

[23] Fukuda M, Shibata S, Shibata N, Hagihara K, Yaguchi H, Osada H, et al Safety comparison of additives in antiglaucoma prostaglandin (PG) analog ophthalmic formulations Clin Ophthalmol. 2013;7:515–20. Epub March 2013. 10.2147/OPTH.S40147 23515900 10.2147/OPTH.S40147 PMC3601024

[24] Rios JD, Shatos MA, Urashima H, Dartt DA. Effect of OPC-12759 on EGF receptor activation, p44/p42 MAPK activity, and secretion in conjunctival goblet cells. Exp Eye Res. 2008;86(4):629–36. Epub 2008/02/26. 10.1016/j.exer.2008.01.007 . 18295205 10.1016/j.exer.2008.01.007

[25] Kleine A, Kluge S, Peskar BM. Stimulation of prostaglandin biosynthesis mediates gastroprotective effect of rebamipide in rats. Dig Dis Sci. 1993;38(8):1441–9. Epub 1993/08/01. . 8393757 10.1007/BF01308601

[26] Higuchi K, Arakawa T, Nebiki H, Uchida T, Fujiwara Y, Ando K, et al Rebamipide prevents recurrence of gastric ulcers without affecting Helicobacter pylori status. Dig Dis Sci. 1998;43(9 Suppl):99S–106S. Epub 1998/09/30. . 9753234

[27] Yokoi N, Kinoshita S. Clinical evaluation of corneal epithelial barrier function with the slit-lamp fluorophotometer. Cornea. 1995;14(5):485–9. Epub 1995/09/01. . 8536461

[28] Fukuda M, Sasaki H. Effects of Fluoroquinolone-Based Antibacterial Ophthalmic Solutions on Corneal Wound Healing. J Ocul Pharmacol Ther. 2015;31(9):536–40. Epub 2015/07/18. 10.1089/jop.2014.0118 . 26186125 10.1089/jop.2014.0118

